# Accelerated pacing rates to improve exercise tolerance, quality of life, and arrhythmia burden in patients with evidence of heart failure with preserved ejection fraction: The PACE-UP rationale and design

**DOI:** 10.1016/j.hroo.2026.07.006

**Published:** 2026-07-13

**Authors:** Jackson O. Howie, Jenelle K. Dziano, Jonathan P. Ariyaratnam, Mehrdad Emami, Shaun Evans, Melissa E. Middeldorp, Alexis Puvrez, Genevieve T. Kenny, Prashanthan Sanders, Adrian D. Elliott

**Affiliations:** Centre for Heart Rhythm Disorders, Adelaide University, and Royal Adelaide Hospital, Adelaide, Australia

**Keywords:** Heart failure with preserved ejection fraction, Permanent pacemaker, Accelerated pacing, Exercise capacity, Peak oxygen consumption (VO_2peak_)

## Abstract

**Background:**

Patients with permanent pacemakers often present with early or established heart failure with preserved ejection fraction (HFpEF). Increasing the lower pacing rate has demonstrated improvements in quality of life and patient physical activity, while lowering the burden of atrial arrhythmias. However, the impact on objective measures of exercise tolerance and cardiac function is not well established.

**Objective:**

This study aims to assess the impact of an accelerated lower rate setting on peak oxygen consumption in patients with stage B/C HFpEF.

**Methods:**

The Accelerated Pacing Rates to Improve Exercise Tolerance, Quality of Life, and Arrhythmia Burden in Patients with Evidence of Heart Failure with Preserved Ejection Fraction study is a multicenter, prospective, 2-arm randomized controlled trial recruiting 160 participants. Participants will be randomized to accelerated pacing rate (75 beats per minute) or usual care (60 beats per minute), with follow-up assessments at 4 and 52 weeks after randomization.

**Results:**

The primary endpoint is peak oxygen consumption obtained during cardiopulmonary exercise testing at 52 weeks after randomization. Secondary endpoints include change in N-terminal pro-B-type natriuretic peptide, Minnesota Living With Heart Failure Questionnaire, device-detected atrial arrhythmias and physical activity, resting and exercise transthoracic echocardiography, and Montreal Cognitive Assessment.

**Conclusion:**

This trial will be the first randomized trial to assess the impact of a high lower rate pacing on objective exercise tolerance and cardiac function in patients with stage B/C HFpEF. We anticipate the findings to improve our knowledge and guide pacemaker programming for patients with HFpEF.

**Key Findings**

▪ The Accelerated Pacing Rates to Improve Exercise Tolerance, Quality of Life, and Arrhythmia Burden in Patients with Evidence of Heart Failure with Preserved Ejection Fraction (PACE-UP) trial is the first randomized trial to evaluate the effect of accelerated pacing on objectively measured exercise tolerance in stage B/C heart failure with preserved ejection fraction (HFpEF).
▪ A total of 160 participants will be randomized to accelerated pacing at 75 beats per minute or usual care, with 4- and 52-week follow-up.
▪ The primary endpoint is change in peak oxygen consumption obtained from cardiopulmonary exercise testing, an objective measure of exercise tolerance.
▪ Secondary endpoints include changes in natriuretic peptides, heart failure symptoms, atrial arrhythmia burden, device-detected physical activity, cardiac structure and function, and cognition.
▪ PACE-UP will provide mechanistic insights into heart rate modulation in HFpEF and may inform future pacemaker programming strategies.

## Introduction

Worldwide, an estimated 64.3 million individuals have heart failure (HF), and approximately half have HF with preserved ejection fraction (HFpEF).^1^^,^^2^ HF is associated with impaired quality of life and increased morbidity and mortality.^3^ Outcomes remain poor, with a 5- and 10-year survival of 56.7% (54.0%–59.4%) and 34.9% (24.0%–46.8%), respectively.^4^

Permanent pacemakers (PPMs) are common, with sinus node dysfunction accounting for most device implants (42.3%).^5^ Given the advanced age and high burden of comorbidities in this population, HFpEF is prevalent among patients with a PPM, affecting approximately one-quarter of patients (22.6%).^6^

### Pathophysiology

HFpEF is heterogeneous involving both cardiac and noncardiac impairments.^7^^,^^8^ Central to HFpEF is abnormal diastolic function, resulting from impaired relaxation and compliance, leading to increased filling pressures.^9^, ^10^, ^11^ Asymptomatic patients with early evidence of HFpEF present with relatively mild cardiac remodeling and impairments in diastolic function, with elevated filling pressures observed at rest or during exercise (stage B HFpEF).^9^^,^^12^ With disease and symptom progression (stage C HFpEF), the rate and extent of early left ventricular (LV) relaxation are decreased, shifting a greater proportion of LV filling into late diastole. This results in LV filling that is reliant on elevated left atrial (LA) pressures to contribute to LV diastole.^9^ Elevated filling pressures at rest and during exercise are cardinal to HFpEF, with sustained exposure resulting in LA structural and functional remodeling, predisposing patients to atrial fibrillation (AF).^12^^,^^13^ LA cardiomyopathy is common in HFpEF, with individuals demonstrating significantly reduced LA compliance, reservoir function, and contractility, all of which are worsened by increasing AF burden.^13^

### Therapeutic options

Therapeutic options for HFpEF are limited. Traditional management has focused on managing fluid and salt restrictions, diuretic therapy, and treatment of comorbidities.^14^^,^^15^ In response to the EMPEROR-Preserved,^16^ PRESERVED-HF,^17^ and DELIVER^18^ trials, sodium–glucose cotransporter-2 inhibitors received a class 1a recommendation for HFpEF.^8^^,^^19^ More recently, glucagon-like peptide-1 (GLP-1) receptor agonists have emerged to reduce adverse cardiovascular events in patients with diabetes and overweight/obesity.^20^ The STEP-HFpEF trial explored the use of GLP-1 in patients with HFpEF, with semaglutide effectively reducing HF symptoms and physical limitations, with greater weight loss than placebo.^20^

Given the pathophysiological significance of elevated filling pressures in HFpEF, both pharmacologic and interventional therapies that target this mechanism have been investigated, with varying degrees of success. Administration of sodium nitrate effectively reduced biventricular filling pressures and pulmonary artery pressures at rest and during exercise.^21^ However, more recent meta-analyses suggest that sodium nitrate has no impact on overall exercise capacity.^22^ Similarly, interatrial shunt devices have been hypothesized to reduce filling pressures by unloading the LA. However, the REDCUE LAP-HF II trial did not find any reduction in HF events or improvement of health status.^23^

### Heart rate modulation in HFpEF

Although heart rate lowering medication is cornerstone therapy among patients with HF with reduced ejection fraction, there is little evidence of benefit in patients with HFpEF.^24^ A meta-analysis of beta-blockers in HF with reduced, midrange, and preserved ejection fraction demonstrated reduced all-cause and cardiovascular mortality in those with reduced and midrange ejection fraction, with no evidence of benefit in HFpEF.^25^ From large observational data, beta-blocker use in HF with midrange and preserved ejection fraction had no impact on survival and increased the risk of HF hospitalizations, particularly in those with an ejection fraction of >60%.^26^ Similar limitations extend to the use of ivabradine. The EDIFY trial randomized patients with HFpEF to receive ivabradine or placebo with follow-up for 8-months.^27^ Although ivabradine effectively reduced the heart rate by ∼8 beats per minute (bpm), it failed to improve echocardiographic indices of LV filling pressure, 6-minute walk distance, or circulating levels of N-terminal pro-B-type natriuretic peptide (NT-proBNP).^27^ The effect of heart rate lowering medication on exercise tolerance among patients with HFpEF has been explored in several crossover trials. Pal et al^28^ conducted a short-term, placebo-controlled, randomized crossover trial examining the effects of heart rate lowering with ivabradine on exercise capacity in HFpEF. 2 weeks of heart rate reduction led to reduced exercise capacity measured by peak oxygen consumption (VO_2peak_). Supporting the unfavorable effects of heart rate lowering, the PRESERVE-HR trial withdrew beta-blockers in 52 patients with HFpEF and chronotropic incompetence, improving VO_2peak_ by 2.1 mL/kg/min, or 12% of predicted VO_2peak_.^29^

### Pacemaker therapy

PPM implantation is indicated in patients with symptomatic sinus node dysfunction and atrioventricular (AV) block.^30^^,^^31^ Given the wider prevalence of HFpEF in the older, comorbid population, many patients with a PPM also meet the criteria for stage B/C HFpEF.^6^ An analysis of the Acute Decompensated Heart Failure National Registry revealed that, of the 13,881 patients with HFpEF included, 3136 (22.6%) had a PPM.^6^

### Refining pacemaker settings in HFpEF

Accelerated lower rate settings in patients with stage B/C HFpEF may provide functional and clinical benefit through an immediate leftward shift in the pressure-volume relationship (Fig. 1).^32^^,^^33^ Elevated lower rate pacing reduces LV end-diastolic volume, avoiding an exponential rise in LV end-diastolic pressure seen at lower heart rates.^32^ Up to 125 bpm, relaxation kinetics improve, reducing the exposure to myocardial stiffness typical of HFpEF.^34^ This promotes early diastolic filling by suction and alleviates overall filling pressure.^34^ With prolonged exposure to an elevated heart rate and lower filling pressures, reverse remodeling may reduce LV wall thickness and mass-to-volume ratio.^35^

**Figure 1:**
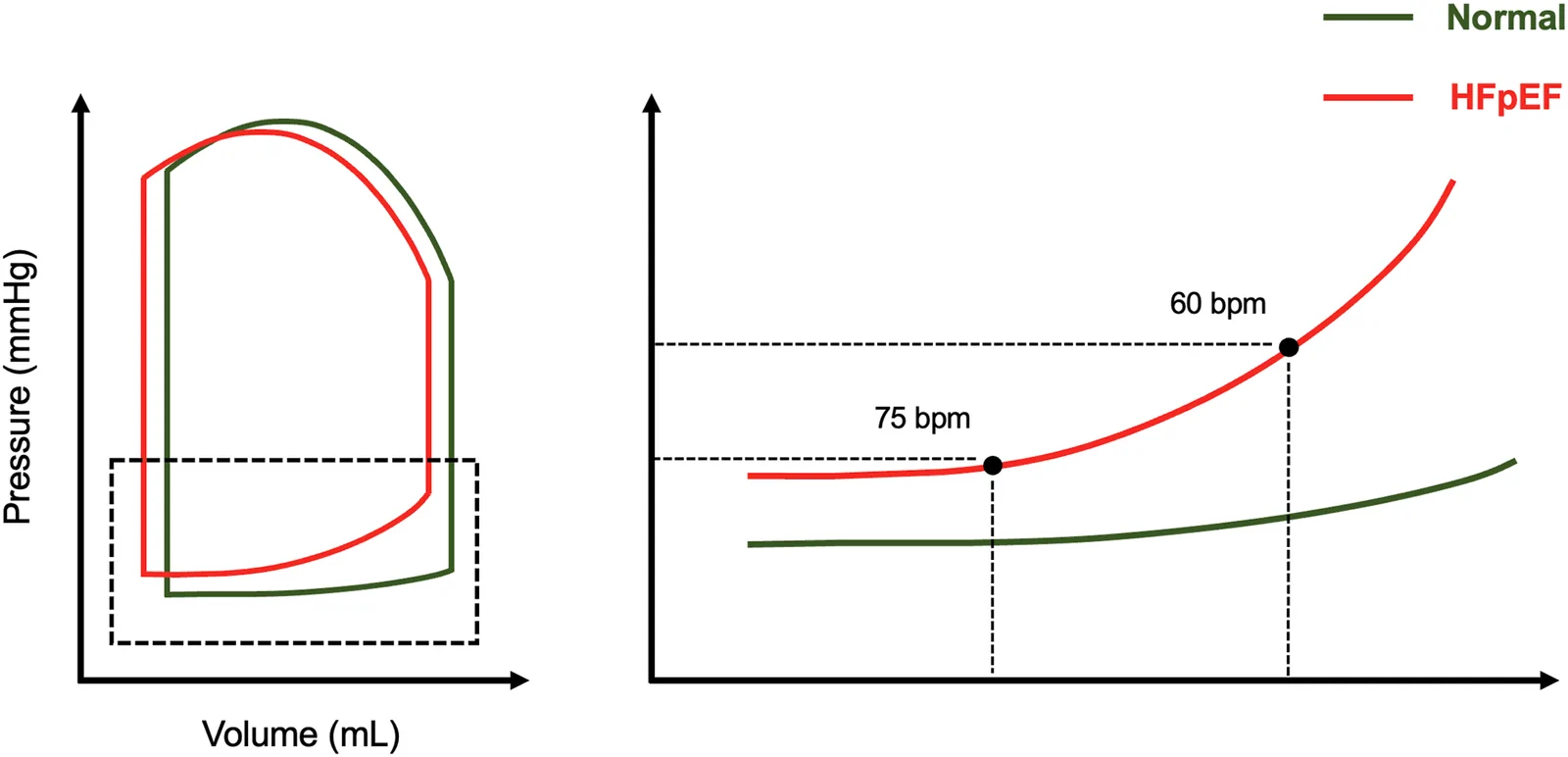
Heart rate modulation effects on the pressure-volume loop in a normal heart and in HFpEF. *Left:* The pressure-volume loop in a normal heart (*green*) and in HFpEF (*red*). The HFpEF loop is shifted upward, resulting in elevated LV filling pressures at equivalent volumes. *Right:* In HFpEF, the steep, upward-shifted LV pressure-volume loop means that small increases in the LV end-diastolic volume result in disproportionate increases in filling pressure. At accelerated pacing rates (75 bpm), the LV end-diastolic volume is reduced, shifting the curve leftward and downward, substantially reducing LV filling pressures. bpm = beats per minute; HFpEF = heart failure with preserved ejection fraction; LV = left ventricular.

In a porcine model of concentric hypertrophy and fibrosis, increased heart rates (150 bpm) were effective in reducing LV wall thickness, increasing LV volumes, decreasing myocardial fibrosis, and improving LV compliance.^36^ The concept of accelerated pacing was examined in humans for safety and feasibility in a small cohort (n = 10) with diastolic dysfunction.^37^ Pacing-induced elevated heart rates (100 bpm) for 5 hours at night were tested over 4 weeks and found to be safe and generally well tolerated.^37^ None of the patients developed signs or symptoms of HF, LV dysfunction, or arrhythmia.^37^ To determine the effect on quality of life as measured by the Minnesota Living With Heart Failure Questionnaire (MLHFQ), 6-minute walk distance, and NT-proBNP, 20 patients with diastolic dysfunction had their PPM lower rate elevated to 80 bpm for 4 weeks, before reversal to their previous lower rate setting.^38^ The elevated pacing rate improved 6-minute walk distance and quality of life. NT-proBNP improvements were limited to those with a QRS of ≤150 ms.^38^ These changes reversed once the PPM lower rate setting were returned to their previous setting.^38^

The myPACE trial was the first randomized trial to implement an accelerated lower rate pacing strategy in patients with a preserved LV ejection fraction. It enrolled 107 patients with stage B/C HF and a PPM. Patients in the intervention arm received personalized accelerated pacing (mean heart rate 75 bpm), with usual care (60 bpm) provided to the control arm.^33^ The trial evaluated quality of life, NT-proBNP, and PPM-detected physical activity, AF, and adverse events. Patients were followed up for 1 year, with accelerated pacing demonstrating improved HF symptoms and physical activity alongside a reduction in arrhythmia burden and NT-proBNP.^33^

The benefits of elevated pacing rates seem to be confined to the resting rate. Despite a high prevalence of chronotropic incompetence in patients with HFpEF,^39^^,^^40^ increased exercising heart rate does not translate to improvements in exercise tolerance. The RAPID-HF randomized crossover trial implanted PPMs in patients with symptomatic HFpEF and chronotropic incompetence to improve exercise heart rate response.^41^ Although PPM implantation effectively increased early and peak exercise heart rate, it failed to improve oxygen uptake at anaerobic threshold.^41^ The RAPID-HF investigators hypothesized that these findings may be caused by a reduction in exercising stroke volume with elevated heart rates, subsequently limiting cardiac output, as shown in previous studies of individuals without HFpEF.^42^

In patients with symptomatic AF undergoing catheter ablation, the role of accelerated pacing has been investigated in a combined clinical-computational study. Investigators studied the relationship between accelerated pacing rates and LA pressure from invasive hemodynamic measurement. In addition, a computational model of virtual patients with HFpEF would determine the effects of accelerated pacing and sequential pacing under controlled in silico conditions. Of the 75 patients included, 65% had a high likelihood of HFpEF (H_2_FPEF ≥5.0) and 37% had an elevated mean LA pressure of ≥15 mm Hg. At the individualized optimal accelerated pacing rate (median heart rate 100 bpm), invasively measured mean LA pressure reduced by −2.3 mm Hg, but induced decremental AV conduction delay. As pacing rates approached 130 bpm, mean LA pressure increased, surpassing resting pressures in sinus rhythm. These findings were confirmed by computer simulations, additionally revealing that the optimal pacing rate may be further optimized with AV sequential pacing.^43^

Although the myPACE trial demonstrated an improvement in HF symptoms with accelerated pacing, further assessment of functional outcomes including exercise tolerance is warranted. VO_2peak_ assessed with cardiopulmonary exercise testing provides an objective assessment of exercise tolerance in response to therapy among patients with HFpEF.^44^ Therefore, we hypothesize that accelerated pacing in patients with evidence of stage B/C HFpEF will increase VO_2peak_, alongside improvements in NT-proBNP, MLHFQ, device-detected arrhythmia burden and physical activity, resting and exercise transthoracic echocardiography, and Montreal Cognitive Assessment (MoCA).

## Objectives

The primary objective of the Accelerated Pacing Rates to Improve Exercise Tolerance, Quality of Life, and Arrhythmia Burden in Patients with Evidence of Heart Failure with Preserved Ejection Fraction (PACE-UP) trial is to compare accelerated PPM lower rate settings with standard lower rate settings on VO_2peak_ in patients with stage B/C HFpEF at 52 weeks after randomization. Secondary outcomes include change in NT-proBNP, MLHFQ, device-detected atrial arrhythmia burden and physical activity, resting and exercise transthoracic echocardiography, and MoCA.

## Methods

### Trial design and oversight

The PACE-UP trial is a multicenter, prospective, 2-arm randomized controlled trial recruiting 160 participants from 5 hospitals in Adelaide, South Australia. Participants will be identified from attendance at pacing clinics and referred to the Centre for Heart Rhythm Disorders. Participants will be randomized 1:1 to accelerated pacing rate (Hi-PACE) or usual care (Lo-PACE), performing assessments at baseline and at 4 and 52 weeks after randomization (Fig. 2).

**Figure 2:**
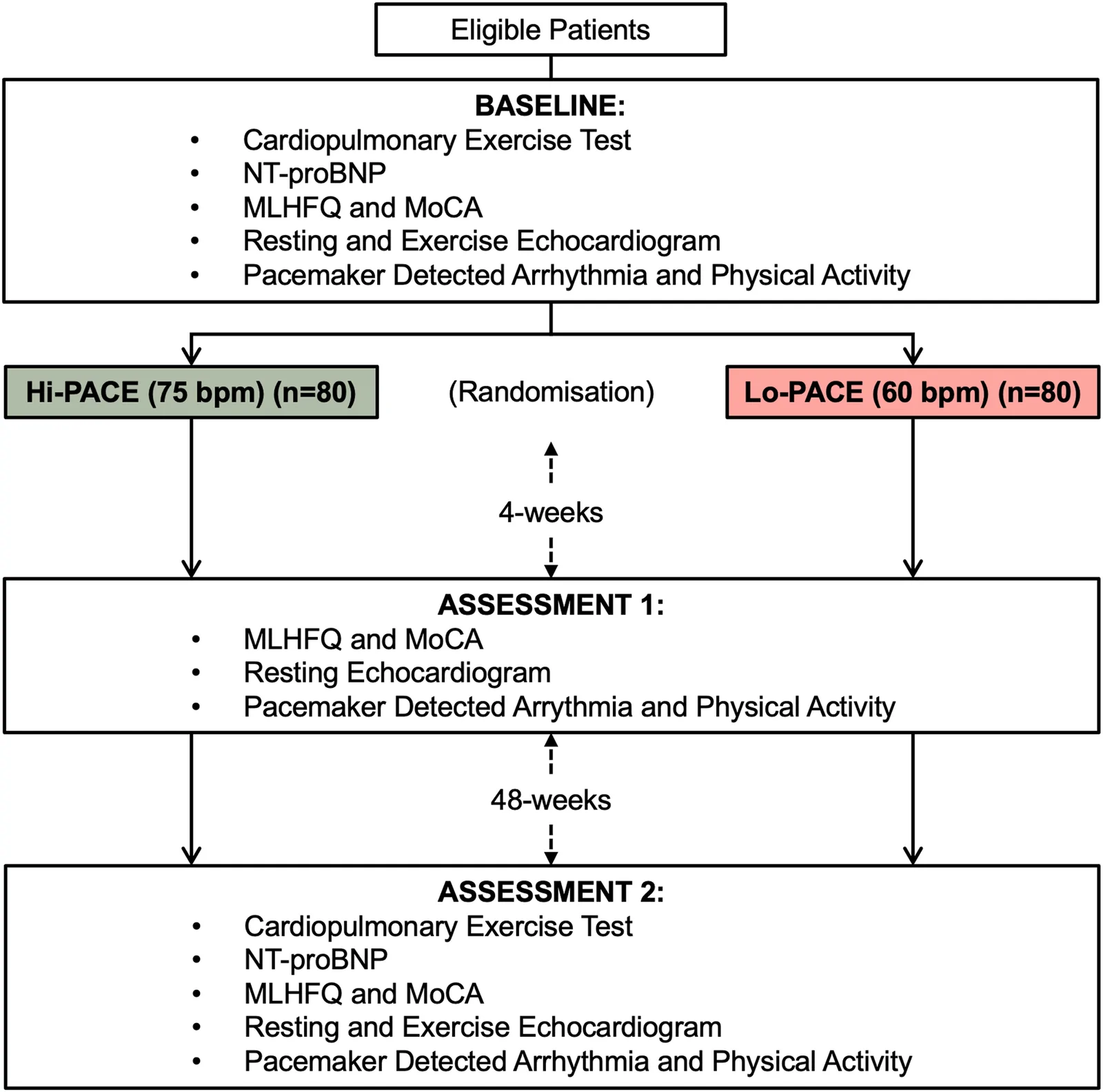
Participant pathway flowsheet. bpm = beats per minute; MLHFQ = Minnesota Living With Heart Failure Questionnaire; MoCA = Montreal Cognitive Assessment; NT-proBNP = N-terminal pro-B-type natriuretic peptide.

The trial is conducted in accordance with the principles of the Declaration of Helsinki and the Good Clinical Practice guidelines of the International Conference on Harmonisation. Ethical and governance approval has been obtained from the relevant ethical committees for the respective recruiting sites. All participants must provide a written informed consent before inclusion. The trial is funded by a grant awarded from the National Heart Foundation of Australia with award ID 107339. The trial is registered at the Australian New Zealand Clinical Trials Registry with trial ID ACTRN12623001187639.

### Study population

Adults with a PPM meeting criteria for HFpEF will be eligible. The diagnosis of HFpEF will be made according to the Heart Failure Association of the European Society of Cardiology or the H_2_FPEF scoring systems.^45^^,^^46^ The Heart Failure Association of the European Society of Cardiology diagnostic algorithm includes functional and morphologic echocardiographic parameters, together with NT-proBNP from pathology. Patients with a score of ≥5 points are considered to have HFpEF and eligible for inclusion.^45^ The H_2_FPEF score includes clinical and echocardiographic parameters, with a score of ≥6 points considered HFpEF and eligible for inclusion.^46^ Further details of the HFpEF scoring systems are included in the supplement.

Eligible participants must have a PPM implanted for sinus node disease or AV node disease. To minimize the potential adverse effects of right pacing on right ventricular pacing–induced dyssynchrony, participants with conventional right ventricular pacing should have minimal right ventricular pacing (<20%) and a paced QRS of <150 ms from preexisting clinical electrocardiograms (ECGs). Participants with impaired AV node conduction with His bundle, left bundle branch area pacing, or biventricular pacing will be eligible irrespective of pacing burden (Table 1).

**Table 1:** Participant inclusion and exclusion criteria

Inclusion criteria	Exclusion criteria
• Adults 18–90 y old with a pacemaker • Stage B/C heart failure with preserved ejection fraction according to the HFA-PEEF or H_2_FPEF diagnostic scoring criteria^45^^,^^46^ • Sinus node dysfunction with intact AV node conduction, minimal RV pacing (<20%), and a paced QRS of <150 ms Or • Impaired AV node conduction with His bundle, left bundle branch area pacing, or biventricular pacing • Subject is expected to remain available for follow-up visits.	• Left ventricular ejection fraction of <45% • Inability to participate in exercise testing owing to musculoskeletal disease or other active diseases • Myocardial infarction within the past 12 mo • Infiltrative cardiomyopathy • Hypertrophic cardiomyopathy • More than moderate valvular stenosis or regurgitation • Aortic valve replacement in the past 1 y • Significant primary pulmonary disease on home oxygen • Uncontrolled hypertension as defined by a BP of >160/100 mm Hg on 2 measurements ≥15 min apart • Creatinine of >2.5 or hemoglobin of <8 g/dL • Pregnancy • Life expectancy of <1 y

AV = atrioventricular; BP = blood pressure; HFpEF = Heart failure with preserved ejection fraction; H_2_FPEF = clinical risk score estimating probability of HFpEF; RV = right ventricle.

### Randomization and blinding

Participants will be randomized 1:1 using a computer-generated Web-based randomization schedule. Participants will remain blinded to treatment allocation. Clinicians responsible for PPM programming will not be blinded owing to the nature of the intervention. Adjudication of the primary and secondary outcomes will be performed at Adelaide University by clinical scientists, blinded to group allocation. Where there is disagreement, a third opinion will be sought from an individual with training in analysis of cardiopulmonary exercise testing. It is acknowledged that the resting ECG trace may allow examiners to suspect group allocation. In addition, participants with a smart watch or similar technologies may also suspect allocation. To minimize bias, all endpoint assessments will follow predefined objective criteria outlined for each outcome. All key secondary outcomes will be assessed by investigators without knowledge of treatment allocation.

### Intervention (Hi-PACE) arm

Participants randomized to the Hi-PACE arm will have their PPM device adjusted to a lower rate setting of 75 bpm. A fixed rate (in comparison to a personalized allometric rate) of 75 bpm was selected based on data that the intrinsic resting heart rate in older adults aged 71–80 years is 74.2 bpm^47^ and that the myPACE individualized pacing algorithm achieved a median programmed rate of 75 bpm.^33^ AV delay and rate response will be left to operator discretion. The pacing strategy will aim to achieve accelerated atrial pacing with minimal right ventricular pacing. Alternatively, patients with conduction system pacing may undergo accelerated pacing through those modalities. Adjustment of the lower rate setting will be performed by a cardiac physiologist during PPM interrogation. The cardiac physiologist modifying PPM settings and the supervising cardiologist will not be involved in the assessment of any study outcomes.

### Usual care (Lo-PACE) arm

The standard PPM lower rate setting is 60 bpm. Participants randomized to the Lo-PACE arm will be maintained at this setting. Rate stabilization and atrial preference pacing will be programmed off to ensure that the heart rate remains at 60 bpm. AV delay and rate response will be left to operator discretion. To facilitate participant blinding, participants in the Lo-PACE arm will still undergo a baseline PPM interrogation and identical follow-up as the intervention arm.

### Primary endpoint

The primary endpoint for the PACE-UP trial is VO_2peak_ obtained during cardiopulmonary exercise testing at 52 weeks after randomization. The rationale for selection of VO_2peak_ is its frequent use as an endpoint in HFpEF clinical trials,^28^, ^48^, ^49^, ^50^, ^51^ permitting comparison with other therapeutic effects.

### Secondary endpoints

Key secondary endpoints include (1) change in NT-proBNP, (2) quality of life as measured by the MLHFQ, and (3) atrial arrhythmia burden at 12 months after randomization. Other secondary endpoints include device-detected physical activity, LA and LV structure and function from resting and exercise echocardiography, and cognition as measured by MoCA (Table 2).

**Table 2:** Follow-up schedule

Timeline of assessments	Enrolment	4 wk	52 wk
Clinical review	**✓**		**✓**
Cardiopulmonary exercise test	**✓**		**✓**
NT-proBNP	**✓**		**✓**
MLHFQ	**✓**	**✓**	**✓**
Device-detected arrhythmia burden	**✓**	**✓**	**✓**
Device-detected physical activity	**✓**	**✓**	**✓**
Resting echocardiography	**✓**	**✓**	**✓**
Exercise echocardiography	**✓**		**✓**
MoCA	**✓**	**✓**	**✓**

MLHFQ = Minnesota Living With Heart Failure Questionnaire; MoCA = Montreal Cognitive Assessment; NT-proBNP = N-terminal pro-B-type natriuretic peptide.

### Cardiopulmonary exercise testing

Cardiopulmonary exercise testing will be performed on an upright cycle ergometer (Lode Corival, Lode B.V.). Oxygen consumption (VO_2_) and carbon dioxide production will be analyzed continuously and averaged over 20-second intervals using a calibrated metabolic cart (Vyntus CPX, Vyaire Medical). Heart rate will be measured using a 12-lead ECG, alongside systolic and diastolic blood pressure and rating of perceived exertion recorded in 3-minute intervals. Participants began cycling at 20 W, increasing by 10 W/min. Peak exercise was defined as the point at which the participant stopped testing from fatigue or symptoms and a respiratory exchange ratio (RER) of >1.05. The VO_2peak_ will be identified as the highest achieved VO_2_ throughout exercise.

### NT-proBNP

NT-proBNP will be quantified from venous blood samples obtained at baseline and 52 weeks after randomization. The cardiac rhythm at the time of sampling will be recorded.

### Pacemaker interrogation

Pacemaker-detected atrial arrhythmia burden throughout the 52-week study period will be compared between groups, after adjudication by a cardiac electrophysiologist blinded to treatment allocation. Using PPM in-built accelerometers, we will also compare device-detected physical activity (hours of activity per day) throughout the 52 weeks.

### HF symptoms and cognition

The MLHFQ is an extensively validated tool to assess physical symptoms, functional limitations, and psychosocial effects of HF. The MLHFQ will be assessed at baseline and at 4 and 52 weeks after randomization. The MoCA is a commonly used clinical and research tool for the early detection of mild cognitive impairment. Cognitive impairment is common in elderly HF patients and often goes undetected.^52^ Including the MoCA allows for the assessment of cognition and whether accelerated PPM affects cognition in HF patients. The MoCA will be assessed at baseline and at 4 and 52 weeks after randomization.

### Resting and exercise echocardiogram

Resting transthoracic echocardiography will be performed at baseline and at 4 and 52 weeks after randomization in accordance with clinical guidelines.^53^^,^^54^ Exercise echocardiography will be performed at baseline and 52 weeks after randomization. The exercise echocardiography protocol will be performed on a dedicated tilting cycle ergometer (Ergoselect 1200, Ergoline). The protocol consists of 3 stages: (1) rest, (2) target heart rate of 90 bpm, and (3) target RER of 1.00. To achieve this, participants will pedal at a constant 60 rpm, with the power increasing in 10 W increments to achieve the target stage. Transthoracic echocardiographic imaging will be performed at each stage with focused apical 2- and 4-chamber views of the LA and LV. Measurements obtained at each stage include mitral inflow velocity (E), mitral annular velocities (e’) (average E/e′ will be calculated as the mean septal and lateral E/e’), tricuspid regurgitation maximum velocity, LV global longitudinal strain, LV ejection fraction, LA emptying fraction, and LA strain. Booster pump and conduit strain will not be obtained at the third stage because fusion is commonly seen in the reservoir and booster pump phases. All echocardiographic measurements will be averaged over 3 cardiac cycles in sinus rhythm and 6 cycles in AF, then independently verified by a second reviewer. Continuous gas analysis will be performed throughout the protocol, allowing for the measurement of VO_2_, carbon dioxide production, and RER at each exercise stage. Stroke volume and cardiac output will be calculated from echocardiographic measurement of LV outflow tract velocity-time integral with simultaneous gas analysis data, enabling assessment of central and peripheral limitations throughout exercise (Table 3).

**Table 3:** Primary and secondary endpoints

Primary endpoint
• Change in VO_2peak_ at 52 wk after randomization
Secondary endpoint
• Change in plasma NT-proBNP at 52 wk
• Change in MLHFQ score at 52 wk
• Device-detected atrial arrhythmia burden over 52 wk
• Device-detected physical activity over 52 wk
• Change in LA and LV structure and function at 52 wk
• Change in MoCA at 52 wk

LA = left atrial; LV = left ventricular; MLHFQ = Minnesota Living With Heart Failure Questionnaire; MoCA = Montreal Cognitive Assessment; NT-proBNP = N-terminal pro-B-type natriuretic peptide; VO_2peak_ = Peak oxygen consumption.

### Sample size calculation and statistical analysis

In a crossover trial of beta-blocker withdrawal in HFpEF participants, a VO_2peak_ of 12.4 ± 2.9 mL/kg/min was observed at baseline.^29^ We anticipate that a between-group difference of 1.5 mL/kg/min would be clinically meaningful and exceeds the typical intrasubject variability in VO_2peak_.^55^^,^^56^ Assuming 5% noncompliance/crossover, a sample size of 160 participants will be recruited (alpha = 0.05; beta = 0.20). The primary outcome (VO_2peak_) will be analyzed using a linear mixed-effects model with fixed effects for treatment group, time, and their interaction, and a random intercept for each participant to account for repeated measures. Missing data will be handled using multiple imputation under the missing-at-random assumption, with sensitivity analyses performed. Results will be reported as mean differences with 95% confidence intervals, and statistical significance at *P* < .05. Secondary endpoints will be analyzed using similar mixed-effects models. Categorical outcomes will be compared using χ^2^ or Fisher’s exact tests. All analyses will follow the intention-to-treat principle and will be conducted using R version 2025.5.1.513.

## Discussion

The therapeutic options for HFpEF remain limited. Although recent medical therapy trials have demonstrated benefit with sodium–glucose cotransporter-2 inhibitors and GLP-1 receptor agonists, many patients continue to experience impaired functional capacity and HF symptoms.^20^^,^^57^ Accelerated PPM lower rate settings have emerged as a novel approach to address the hemodynamic abnormalities central to HFpEF, with the myPACE trial demonstrating an improvement in HF symptoms, physical activity, arrhythmia burden, and cardiac biomarkers.^33^ However, previous studies were not designed to assess objective measures of exercise capacity, leaving the clinical impact of this strategy uncertain. The PACE-UP trial addresses this gap by evaluating the effects of accelerated pacing on VO_2peak_ from cardiopulmonary exercise testing, alongside the assessment of cardiac remodeling, HF symptoms, cognitive function, and arrhythmia burden. The results of PACE-UP will provide objective evidence on the potential role of heart rate acceleration as a therapeutic strategy in patients with HFpEF.

HFpEF is heterogeneous, contributing to the difficulty in identifying and treating these patients. Accordingly, several phenotypes of HFpEF have been identified.^58^ Most relevant to the PACE-UP trial, the aging phenotype of HFpEF is characterized by older age, female sex, cardiovascular risk factors, systemic changes that eventuate in cardiac remodeling, and elevated biomarkers.^58^^,^^59^ With advancing age, the prevalence of bradyarrhythmia and PPM implantation increases, creating a unique opportunity to selectively increase heart rate.

The patients enrolled in myPACE were older with lower body mass index and more often had AF and coronary artery disease relative to previous medical therapy trials,^20^^,^^57^ suggestive of the aging phenotype of HFpEF. In contrast, patients in GLP-1 receptor agonist trials such as SELECT and STEP-HFpEF were younger and had a higher body mass index, fitting the obese HFpEF phenotype.^58^^,^^59^ With a different phenotype of HFpEF, the mechanism of therapeutic benefit also likely differs. Accelerated pacing targets an immediate reduction in filling pressures by reducing the LV end-diastolic volume, potentially leading to cardiac remodeling.^33^^,^^35^ Given older, less overweight patients regularly present with a PPM, this unlocks a potential new therapeutic option for this phenotype of patients with HFpEF.^6^ The effects of heart rate modification on the obese phenotype of HFpEF are yet to be explored.

### Limitations

We acknowledge several potential limitations with the PACE-UP study. First, we chose not to use a tailored approach to determining the lower rate setting for patients in the intervention group. Although we appreciate that tailoring the heart rate would be appealing, we believe that a fixed target of 75 bpm remains physiologically justified and clinically pragmatic. Intrinsic resting heart rates in older adults aged 71–80 years (an expected age of patients with PPMs) is 74.2 bpm.^47^ The median programmed rate achieved through the myPACE trial individualized approach was 75 bpm, the identical target used in our study.^33^ In addition, a reduction in LA pressure is consistently observed across heart rates of 70–80 bpm, as shown in a recent clinical-computational study.^43^ An additional limitation may be the inclusion of patients with conventional right ventricular pacing and conduction system pacing. Although increasing the pacing rate in patients with conduction system pacing may provide hemodynamic benefit, a large proportion of patients in the community still have conventional pacing systems. Therefore, we chose to include both groups for broader clinical applicability. Finally, our study is not powered for longer-term clinical outcomes including HF events and cardiovascular mortality, which may require further study.

The PACE-UP randomized controlled trial seeks to add to the emerging evidence in favor of accelerated pacing by providing objective measures of exercise capacity (VO_2peak_), a universally used and clinically relevant endpoint in HFpEF trials.^28^, ^48^, ^49^, ^50^, ^51^ In addition, patient-reported outcomes, such as HF symptoms and cognition, determine components of disease that are not fully reflected by physiological measures or level of underlying disease, but remain important to both patients and clinicians.^60^ We anticipate that the findings from PACE-UP will be meaningful, with real clinical implications for this challenging to treat cohort. If successful, findings could provide a new therapeutic strategy for patients with HFpEF and a PPM, particularly those who are older and less overweight, a cohort with few therapeutic options currently available.

## Conclusion

The PACE-UP trial will be the first to determine the effects of accelerated PPM lower rate settings on objectively measured exercise tolerance, alongside natriuretic peptides, quality of life, and atrial arrhythmia burden in patients with stage B/C HFpEF.

## Disclosures

P.S. reports serving on the medical advisory board for Abbott, Medtronic, Boston Scientific, CathRx, and PaceMate. Adelaide University has received on behalf of P.S. research funds from Boston Scientific, Medtronic, Abbott, and Becton Dickinson. All other authors declare that there is no conflict of interest.
