# Supplementary material for: Accelerated pacing rates to improve exercise tolerance, quality of life, and arrhythmia burden in patients with evidence of heart failure with preserved ejection fraction: The PACE-UP rationale and design
Source: Heart Rhythm O2. 2026 Jul 13;7(10):1997–2005. doi: 10.1016/j.hroo.2026.07.006 (PMC13700396; doi:10.1016/j.hroo.2026.07.006)
Supplement: Supplementary file 1 [file mmc1.docx]

**Heart Failure with Preserved Ejection Fraction Diagnosis:**

Participants will be required to meet the criteria for stage B or stage C heart failure with preserved ejection fraction (HFpEF) at enrolment, according to the diagnostic criteria of the Heart Failure Association of the European Society of Cardiology (HFA-PEEF) or the H_2_FPEF scoring system (1, 2).

The HFA-PEFF diagnostic process is a 4-step algorithm, involving (step 1) a pre-test assessment, (step 2) echocardiography and natriuretic peptide score, (step 3) functional testing, and (step 4) final aetiology. Participants will be enrolled based on the score from functional, morphological and biomarker testing (figure 1). Each domain can contribute maximally 2 points, if any major criterion from this domain is positive, or 1 point if no major but any minor criterion is positive. A total score ≥5 points is considered HFpEF and will be included. A score of 4 will undergo diastolic stress testing in the form of exercise echocardiography. Exercise echocardiography should be considered abnormal if average E/e’ ratio at peak stress increases to ≥15, with or without a peak TR velocity >3.4 m/s. An increase only in TR velocity should not be used to diagnose HFpEF. A score of ≤ 3 will not be included within the trial (1) **(figure 1)**.

The H_2_FPEF scoring system utilises clinical characteristics and echocardiographic data for the diagnosis of HFpEF, and discrimination of noncardiac causes of dyspnea. Participants will be assessed based on their weighted score from six variables, with possible score ranging from 0-9. Variables include obesity, atrial fibrillation, age >60 years, treatment with ≥2 antihypertensives, echocardiographic E/e’ ratio >9, and echocardiographic pulmonary artery systolic pressure >35 mm Hg are included, with a total score of ≥6 considered as HFpEF and included in this study (2) **(figure 2).**

**REFERECES:**

1.	Pieske B, Tschöpe C, De Boer RA, Fraser AG, Anker SD, Donal E, et al. How to diagnose heart failure with preserved ejection fraction: the HFA–PEFF diagnostic algorithm: a consensus recommendation from the Heart Failure Association (HFA) of the European Society of Cardiology (ESC). European heart journal. 2019;40(40):3297-317.

2.	Reddy YN, Carter RE, Obokata M, Redfield MM, Borlaug BA. A simple, evidence-based approach to help guide diagnosis of heart failure with preserved ejection fraction. Circulation. 2018;138(9):861-70.

**Figure S1.** The HFA-PEFF Diagnostic Scoring System

BNP = B-type natriuretic peptide; HFpEF = Heart failure with preserved ejection fraction; LA = Left atrial; LV = Left ventricle; NT-proBNP = N-terminal pro–B-type natriuretic peptide.

**Figure S2.** The H_2_FPEF Diagnostic Scoring System
